# Transcription factor induced conversion of human fibroblasts towards the hair cell lineage

**DOI:** 10.1371/journal.pone.0200210

**Published:** 2018-07-06

**Authors:** María Beatriz Duran Alonso, Iris Lopez Hernandez, Miguel Angel de la Fuente, Javier Garcia-Sancho, Fernando Giraldez, Thomas Schimmang

**Affiliations:** 1 Instituto de Biología y Genética Molecular, Universidad de Valladolid y Consejo Superior de Investigaciones Científicas, C/Sanz y Forés 3, Valladolid, Spain; 2 CEXS, Universitat Pompeu Fabra, Parc de Recerca Biomédica de Barcelona, Barcelona, Spain; University of Washington, UNITED STATES

## Abstract

Hearing loss is the most common sensorineural disorder, affecting over 5% of the population worldwide. Its most frequent cause is the loss of hair cells (HCs), the mechanosensory receptors of the cochlea. HCs transduce incoming sounds into electrical signals that activate auditory neurons, which in turn send this information to the brain. Although some spontaneous HC regeneration has been observed in neonatal mammals, the very small pool of putative progenitor cells that have been identified in the adult mammalian cochlea is not able to replace the damaged HCs, making any hearing impairment permanent. To date, guided differentiation of human cells to HC-like cells has only been achieved using either embryonic stem cells (ESCs) or induced pluripotent stem cells (iPSCs). However, use of such cell types suffers from a number of important disadvantages, such as the risk of tumourigenicity if transplanted into the host´s tissue. We have obtained cells expressing hair cell markers from cultures of human fibroblasts by overexpression of *GFI1*, *Pou4f3* and *ATOH1* (GPA), three genes that are known to play a critical role in the development of HCs. Immunocytochemical, qPCR and RNAseq analyses demonstrate the expression of genes typically expressed by HCs in the transdifferentiated cells. Our protocol represents a much faster approach than the methods applied to ESCs and iPSCs and validates the combination of GPA as a set of genes whose activation leads to the direct conversion of human somatic cells towards the hair cell lineage. Our observations are expected to contribute to the development of future therapies aimed at the regeneration of the auditory organ and the restoration of hearing.

## Introduction

Hearing loss is the most prevalent sensorineural deficit in humans, most frequently caused by damage of hair cells (HCs). These are mechanoreceptor cells in the cochlear part of the inner ear and responsible for transducing the information arriving in the form of incoming sound waves to the auditory neurons that connect to the brain. Although a small number of inner ear progenitor cells have been identified in neonatal animals that allow for a certain degree of repair following damage, these appear to be dormant in older individuals, which could explain the observed lack of HC regeneration [1,2]. Approaches that could thus be envisaged towards the restoration of hearing are either the *in vivo* transdifferentiation into HCs of other cell types present in the inner ear (e.g. supporting cells) or transplantation of HC-like cells that have been derived from distinct tissue sources [2,3,4]. HC-like cells have been obtained from embryonic stem cells (ESCs) and induced pluripotent stem cells (iPSCs) [5,6,7,8,9,10,11,12,13]. Although the studies where these cells have been more thoroughly characterized have identified properties of vestibular rather than cochlear HCs [9,14], they set the basis for obtaining their auditory counterparts.

A promising alternative to the ESCs or iPSCs may be the use of patient-derived somatic cells that, although fully differentiated, can be changed into the desired cell fate while bypassing the full reprogramming process typically undergone by iPSCs [15,16,17,18,19]. This strategy has already resulted in the successful transdifferentiation of fibroblasts, hepatocytes, astrocytes and various differentiated blood cell types into other cell lineages (e.g. cardiomyocytes, neurons, macrophages) [20,21,22,23,24,25,26]. For reprogramming, two main routes have been followed, either the induction of epigenetic changes, or the direct conversion of somatic cells into sought-for lineages through the forced expression of lineage-determining factors [16,18,20,26,27,28,29,30]. Epigenetic alterations have been shown to occur following exposure of the cells to chromatin modifiers such as demethylating agents and histone deacetylase inhibitors, or overexpression of transcription factors such as Sox-2 [31] and Oct-4 [24,32]. This results in the transient expression of sets of genes that are associated with a variety of cell lineages. Subsequent culture of these cells under conditions known to drive the emergence of the cell type of interest *in vivo* will then promote their differentiation. On the other hand, overexpression of transcription factors associated with a given lineage is thought to result in the direct conversion of the donor cells, through the recruitment of downstream genes and the activation of the corresponding signalling cascades [22,28,29]. Importantly, this approach has already been successfully applied *in vivo* [2,23,26,33,34].

In the present study, we have adopted a direct conversion approach in order to obtain cells expressing HC markers from cultures of human fibroblasts (hFIBs). In order to do so, and based on the publication by Costa et al. [7], we overexpressed *GFI1*, *Pou4f3* and *ATOH1* (hereafter referred to as GPA) coding sequences in human fibroblast cell cultures. This led to the differentiation of cells expressing HC markers, and our work thus validates the potential of the GPA combination to convert fibroblasts of human origin into cells expressing hair cell markers. To our knowledge, this is the first time that conversion of human somatic cells towards the hair cell lineage is reported.

## Materials and methods

### Culture of hFIBs

hFIB cultures were established from skin biopsies from human donors from whom written consent had been obtained, as described in [35]. The project was approved by the clinical research ethic committee of the University of Valladolid (reference: 2012/15). The cells were routinely passaged 1:3 and grown in standard human fibroblast medium (hFib Med), consisting of Dulbecco´s Modified Eagle Medium (DMEM; Life Technologies, Eugene, OR, USA) supplemented with 1x Glutamax^TM^-I CTS^TM^ (Gibco, Life technologies, Grand Island, NY, USA) and 1x MEM non-essential amino acid solution (NEAA; Merck, Darmstadt, Germany), Penicillin (100U/ml)-Streptomycin (100 μg/ml, Life technologies, Grand Island, NY, USA) and 10% foetal bovine serum (FBS; Gibco). Infection of the cultures was carried out at passage numbers 4–8.

### DNA constructs and preparation of lentiviral particles

The *OCT*4 sequence was removed from the lentiviral plasmid pSIN-EF2-*OCT*4-Pur (Addgene, Cambridge, MA; plasmid 16579, deposited by James Thomson) as an *Spe*I-*Eco*RI fragment and the remaining DNA vector was used to prepare the constructs pSIN-EF2-*Pou4f3*-Pur and pSIN-EF2-*GFI1*-Pur. The former was built as follows: a 1,7kb *Sal*I-*Eco*RI fragment containing the mouse *Pou4f3* ORF (1kb) and a 0,7kb 3´ UTR genomic sequence was excised from pRK5KS-*Brn3c* (kindly provided by Mengqing Xiang; Piscataway, NJ, USA) and cloned into a pCDNA3 vector; this sequence was subsequently subcloned as an *Xba*I-*Eco*RI fragment into the *Spe*I/*Eco*RI-digested pSIN-EF2-Pur vector. In order to build the pSIN-EF2-*GFI*1-Pur construct, the sequence coding for *GFI1* was amplified from the human cDNA clone SC126131 (OriGene Technologies, Rockville, MD, USA) using PCR primers SpeI-GFI1-F (5´-AACTAGTGCCGGACCACCATGC-3´) and ERI-GFI1-R (5´- GGAATTCATTTGAGCCCATGCTG-3´), carrying an *Spe*I and an *Eco*RI sequence, respectively, and Platinum Taq (Invitrogen, Carlsbad, CA, USA); amplification conditions were: 1 cycle at 95° for 5 minutes; 30 cycles at 94°C for 30 seconds, 53°C for 45 seconds, and 72°C for 2 minutes; 1 cycle at 72°C for 10 minutes. The PCR fragment was purified and its sequence confirmed, and subsequently digested with *Spe*I and *Eco*RI and cloned into the *Spe*I/*Eco*RI-digested pSIN-EF2-Pur vector. The pLV-*ATOH1*-IRES-*eGFP* construct was obtained by excising a *Bam*HI-*Xho*I fragment containing the *ATOH1* coding sequence from the pRV-IRES-*MATH1* (provided by Micaela Gallozzi) and subcloning it into the lentiviral vector pLV-IRES-*eGFP*, kindly provided by Carlos Vicario Abejón (Madrid, Spain).

Although pSIN-EF2-(*Pou4f3* or *GFI1*)-Pur and pLV-*ATOH1*-IRES-*eGFP* belong to the third-generation of lentiviral transfer vectors [36,37], pSIN-EF2-*Pou4f3*-Pur and pSIN-EF2-*GFI1*-Pur viral particles were obtained by co-transfecting either of these DNAs with the second generation packaging plasmid ps*PAX-2* (Addgene plasmid 12260) and the envelope plasmid pMD2.G (Addgene plasmid 12259) into 293FT cells (Invitrogen), using Lipofectamine^TM^ 2000 (ThermoFisher Scientific, Waltham, MA, USA) and according to manufacturer’s instructions. When preparing pLV-ATOH1-IRES-eGFP lentiviral stocks, the pLV-ATOH1-IRES-eGFP construct was co-transfected with the plasmids pMDLg, pRSV and pMD2.C. The former two are third-generation packaging plasmids and the latter is an envelope plasmid [36,37,38]. Viral supernatants were collected at 48 and 72 hours post-transfection, pooled and concentrated by ultracentrifugation.

Titering of the pSIN-EF2-(*Pou4f3* or *GFI1*)-Pur viral stocks was carried out by incubating HeLa cells (plated the previous day at 2x10^5^ cells/well on 6-well dishes in DMEM medium supplemented with 1x Glutamax^TM-I^ CTS^TM^, 1x NEAA, penicillin/streptomycin, 1mM sodium pyruvate (Sigma, Merck), and 10% FBS) with serial dilutions (0, 10^−2^, 10^−3^, 10^−4^, 10^−5^ and 10^−6^) of each viral stock, in the presence of 8μg/ml polybrene (Sigma, Merck). Cultures were changed to fresh growth medium the following day and 24 hours later they were exposed to 1μg/ml puromycin (Sigma, Merck). Following 12–14 days in puromycin selection, the cultures were washed with 1x phosphate buffered saline (PBS; ThermoFisher Scientific, Waltham, MA, USA) and stained for 10 minutes in a solution of 1% crystal violet prepared in 10% ethanol. The cultures were thereafter gently washed with 1x PBS and the number of blue-stained colonies was counted in the 10^−5^ and 10^−6^ wells. Lentiviral titer (transforming units per ml (TU/ml)) was calculated as (Number of colonies/volume the cells were transduced in (ml)) x Dilution factor. Viral titers were 2,3–4,7 x 10^7^ and 4,8–6 x 10^6^ TU/ml for pSIN-EF2-*Pou4f3*-Pur and pSIN-EF2-GFI1-Pur stocks, respectively. Titers of pLV-*ATOH1*-IRES-*eGFP* viral preparations were measured by analysing the percentage of GFP-expressing cells in HeLa cultures that had been transduced with the virus 72 hours earlier, following the same protocol as that for the pSIN-LVs. This was carried out by flow cytometric analysis using a Gallios Flow Cytometer (Beckman Coulter, Brea, CA, US); GFP was detected with a blue solid-state laser (488nm) and a 525 BP 40 filter. pLV-*ATOH1*-IRES-eGFP titers were calculated as [(%GFP(+) cells in well x total No of cells infected)/Volume the cells were transduced in] x Dilution factor, and were in the range of 2,7–6 x 10^7^ TU/ml.

### Lentiviral transduction of hFIB cultures and culture of GPA hFIBs

2,2x10^4^ cells were plated on each well in 6-well dishes, in hFib Med. Cultures were infected four days later, at a MOI of 8, 7 and 1,8 with pLV-*ATOH1*-IRES-*eGFP*, pSIN-EF2-*Pou4f3*-Pur, and pSIN-EF2-*GFI1*-Pur lentiviral preparations, respectively, in fresh hFib Med with 8μg/ml polybrene added. The medium was changed to fresh hFib Med 24 hours later. These cultures are hereafter referred to as GPA hFIBs. Transduction efficiencies performed in triplicate samples were 46,72 ± 20,88%, 76,49 ± 11.01%, and 85,93 ± 2,62% for the pLV*ATOH1*-IRES-*eGFP*, the pSIN-EF2*Pou4f3-*Pur and the pSIN-EF2-*GFI1* viruses, respectively.

Following removal of the transduction medium, some cultures were maintained in hFib Med for 12–15 days; others were grown in serum-free medium (SFM), consisting of DMEM:F12 (Gibco), 1x Glutamax, 1x N-2 Supplement (Gibco), and 2x B27 Supplement (Gibco), containing 20ng/ml recombinant human rhEGF (ImmunoTools, Friesoythe, Germany) and 1μM retinoic acid (RA) (Merck). No antibiotic was added to the culture media during differentiation (except puromycin when preparing samples for RNAseq analyses (see below)) and cells were passaged once onto wells and coverslips coated with 0,1% gelatin (Millipore, Merck).

### Preparation of GPA hFIB samples for RNAseq analysis

Four days after lentiviral transduction, cultures grown in SFM containing 20ng/ml EGF and 1μM RA were changed to fresh medium containing 1,5μg/ml puromycin (Sigma, Merck) and maintained in selection for an additional 8 days. RNA was then extracted from these GPA hFIB cultures using the RNeasy Micro kit (QIAGEN, Hilden, Germany), introducing the DNAse treatment step and according to manufacturer´s instructions. RNA libraries were prepared for sequencing using standard Illumina protocols. Mapping of the reads to the GRCh38 reference genome was performed with Star (version 2.5.2) [39]. Generation of count tables and differential expression was done by parsing Star output with edgeR (version 3.2.3) [40]. Gene ontology was performed using PANTHER [41]. RNAseq data have been deposited at GEO with accession number GSE108905.

### Quantitative reverse transcription polymerase chain reaction (qPCR)

Isolation of total RNA from cell cultures was performed using TRIzol® Reagent (Invitrogen), following the manufacturer’s protocol. RNA samples were quantified on a Spectrophotometer ND-1000 (NanoDrop, Thermo Fisher Scientific). One to two micrograms of total RNA was used to synthesize cDNA with the High Capacity cDNA Reverse Transcription Kit (Applied Biosystems, Life Technologies). cDNA samples were amplified on a LightCycler® 480 II (Roche Molecular Diagnostics, Pleasanton, CA, USA) using SYBR® Green PCR Master Mix (Life Technologies). The thermocycling conditions consisted of an initial denaturation step of 10 minutes at 95°C, followed by 40 cycles at 95°C for 15 seconds and 60°C for 1 min. Primers for each of the genes studied in this work were: *GAPDH*, ACACCCACTCCTCCACCTTTG and CATACCAGGAAATGAGCTTGACAA; *MYOSIN VIIA*, CCCATTCTGGAAGCATTTGG and ACTTTCCGAAACGGCTTGAG; *POU4F3*, TTCTCCAGTCTGCACTCTGG and TGCTCATGGTATGGTAGGTGG; *ESPIN* [11], CAGAGTGCAGGACAAAGACAA and GCAGCGTAGTGGATAGGCAG, and murine *Pou4f3*, TTTGATGAGAGCCTGCTGGC and TGCTCATGGTATGGTAGGTGG. Data were analysed using the Software version LCS480 1.5.0.39. Relative levels of mRNA expression were calculated according to the 2^-ΔΔCt^ method, using GAPDH as the housekeeping gene [42].

### Immunocytochemistry

Immunocytochemical analyses were carried out on cultures grown on glass coverslips coated with 0,1% gelatin (Millipore, Merck). Cells were fixed at room temperature in 4% paraformaldehyde (Merck) for 10–15 minutes. Blocking of non-specific binding sites and cell permeabilization were carried out by adding PBT1 solution (PBS containing 0,1% Triton X-100 (Sigma, Merck), 1% BSA (Sigma, Merck) and 5% FBS) for 15 minutes prior to the addition of the primary antibody; this was diluted in PBT1 and left to incubate for 1 hour at room temperature. Primary antibodies used in this work were: polyclonal rabbit antibody against MYOSIN VIIA (1:500; Proteus BioSciences, Inc., Ramona, CA, USA), mouse monoclonal antibody against POU4F3 (1:50; Santa Cruz Biotechnology, Inc., Santa Cruz, CA, US), polyclonal goat antibody against GFI1 (1:50; R&D Systems, Minneapolis, MN, US), polyclonal goat antibody against ANNEXIN A4 (1:50; R&D Systems, Bio-Techne, Minneapolis, MN, USA) and polyclonal rabbit antibody against ESPIN (1:1000; kind gift from Professor James Hudspeth, New York, NY, USA). Following incubation with the primary antibody, the secondary antibody was added, diluted in PBT2 solution (PBS containing 0.1% Triton X-100 and 1% BSA). Secondary antibodies used were Alexa Fluor® 488-conjugated goat anti-mouse IgG and Alexa Fluor® 568-conjugated goat anti-rabbit IgG, and Alexa Fluor® 647-conjugated donkey anti-goat IgG (Invitrogen, Molecular Probes, Eugene, OR, USA). After 1 hour at room temperature, the cultures were mounted on glass slides using Vectashield mounting medium containing DAPI (4’, 6’-diamidino-2-phenylindole) (Vector Laboratories, Inc., Burlingame, CA, USA) to counterstain nuclear DNA. Samples were viewed under an Eclipse 90i fluorescence microscope (Nikon Instruments Europe B.V., Amsterdam, Netherlands). Confocal images of the GPA hFIB cultures were obtained with a Leica DMI 6000B microscope with a TCS SP5 X confocal system and a WLL laser controlled by LAS AF software (Leica, Spain).

### Statistics

During the course of this work, a total of 12 independent experiments were performed using hFIB Med and another 12 using RA&EGF-containing SFM; each experiment consisted of a control culture kept in the corresponding medium all throughout the course of the experiment and a culture grown in the same medium but to which the GPA viral combination had been added. Each culture was passaged once during the length of the experiment and replated as duplicate cultures that were ultimately pooled together in order to extract RNA for qPCR analysis (for these analyses, each sample was loaded onto duplicate wells into the qPCR plate, with a deviation between duplicates of 0,1–0,3 Ct units), as well as on sets of 2–4 duplicate coverslips that were used for immunocytochemical analyses. At least two duplicate cultures were stained in parallel with each antibody. Data are presented as the mean ± SD; the Student´s t-test was used to analyse the significance of any differences between control and treatment groups and also between cultures grown in hFIB Med and those maintained in RA/EGF. Values of p<0,05 were considered to be statistically significant.

## Results

### Effects of GPA overexpression in hFIB cultures

**Fig 1:**
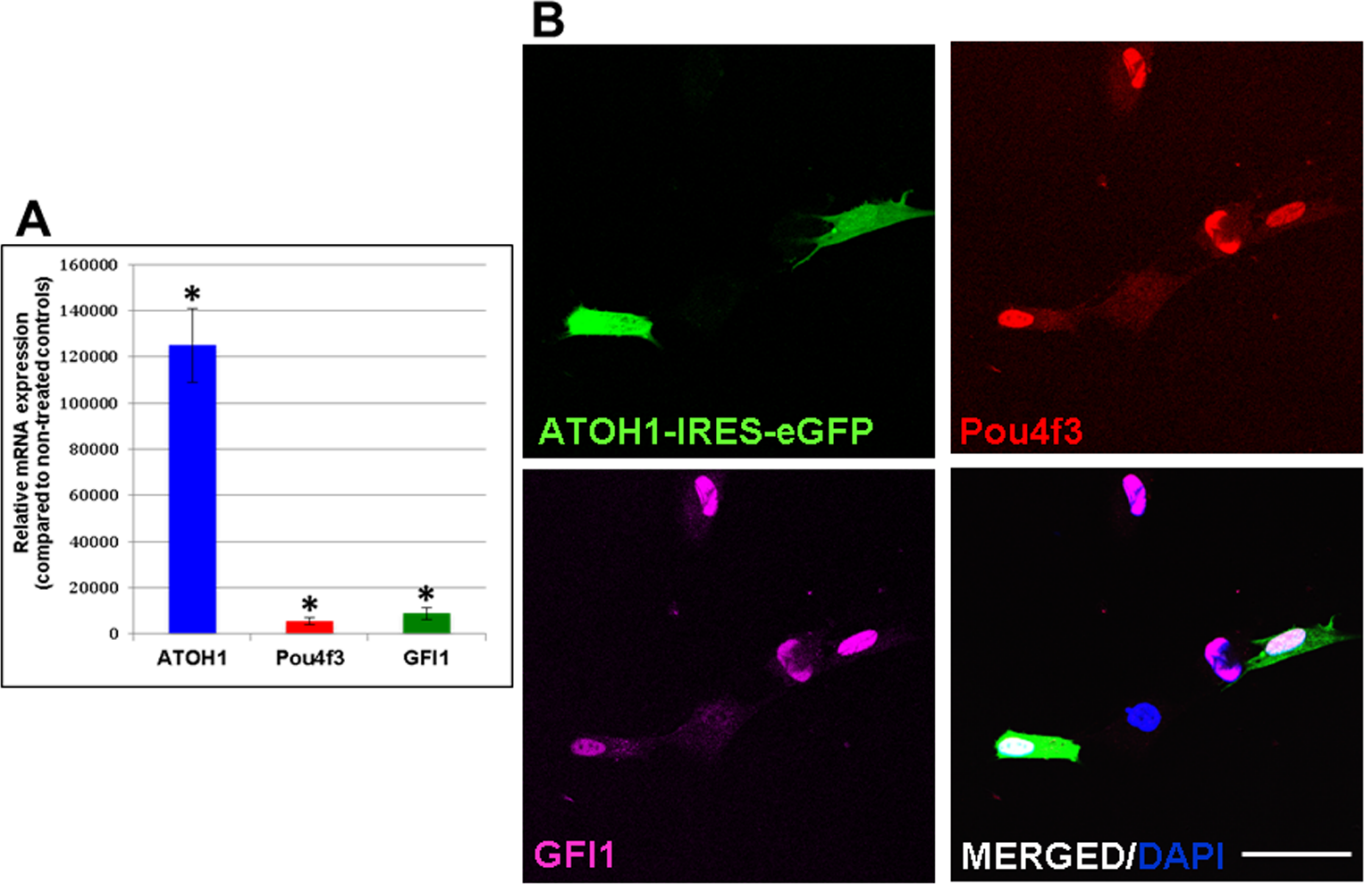
Overexpression of the GPA genes in hFIB cultures. (A) Significantly increased mRNA expression of the genes was observed compared to non-infected control cultures by qPCR analyses. Asterisks: p-value<0,05. (B) GPA protein expression in hFIB cultures simultaneously transduced with pSIN-EF2-*Pou4f3*-Pur, pSIN-EF2-*GFI1*-Pur and pLV-*ATOH1*-IRES-eGFP lentiviruses. Immunostaining of GPA hFIB cultures indicated that a high rate of the GFP(+) cells also expressed the Pou4f3 and the GFI1 proteins. Scale bar: 50μm.

A direct conversion approach was followed [16,17,30], based on the over-expression of *GFI1*, *Pou4f3 and ATOH1* (GPA), three genes whose co-expression has been shown to result in the emergence of HC-like cells in cultures of mESCs [7]. Forced expression of GPA was achieved through infection with lentiviral particles carrying the coding sequences of each of these genes. Overexpression of the three genes was confirmed by qPCR and their coexpression in the transduced cells was analyzed by immunocytochemistry (see Fig 1 and methods). Individual transduction rates were 46,72 ± 20,88%, 76,49 ± 11.01%, and 85,93 ± 2,62% for the LV*ATOH1*-IRES-GFP, the LV*Pou4f3* and the LV*GFI1* viruses, respectively, with 72,81 ± 6,14% of the GFP-positive cells simultaneously expressing both the Pou4f3 and GFI1 proteins (Fig 1B).

**Fig 2:**
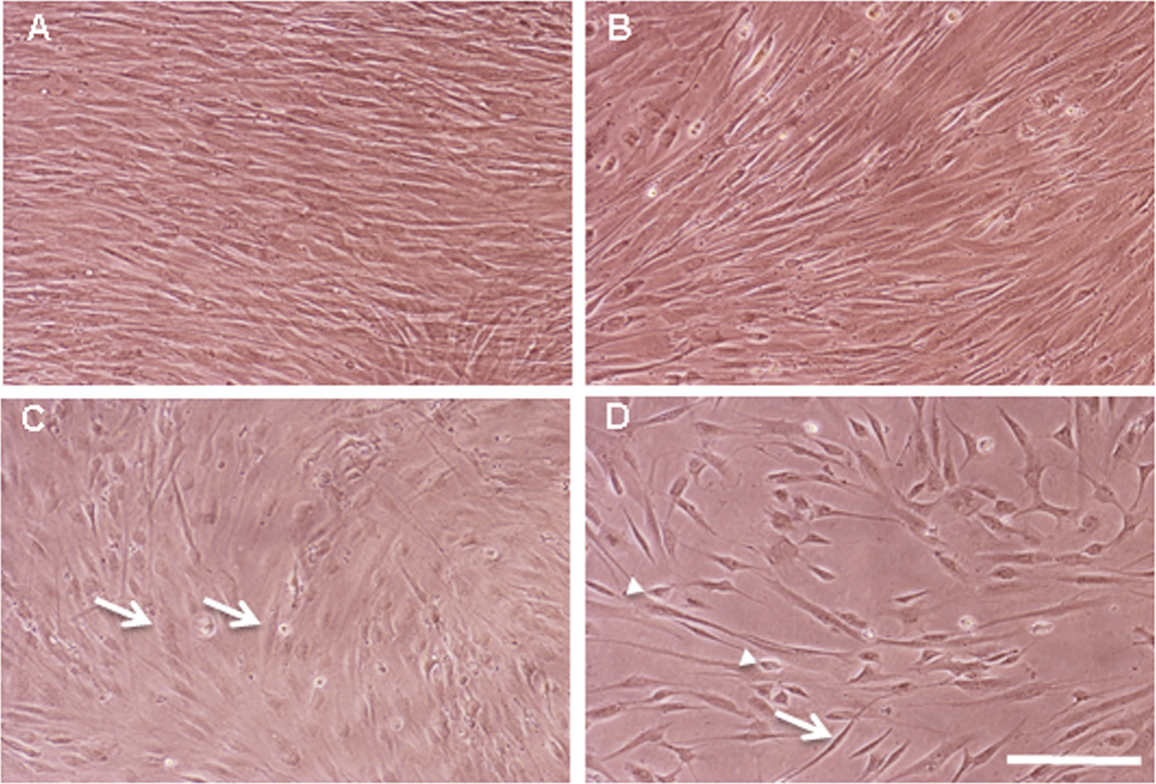
Cultures of hFIBs. Photomicrographs of living hFIB cultures grown in hFIB Med (A and B) or in RA&EGF-containing SFM (C and D) for two weeks. (A and C) Non-infected control cultures; (B and D) GPA hFIBs transduced with lentiviral particles carrying the *GFI1*, *Pou4f3* and *ATOH1* ORFs. Non-infected control cultures (A) and GPA transduced hFIBs (B) grown in hFIB Med showed densely packed fusiform cells, while in the presence of RA&EGF-containing SFM (C) a lower cell density was observed with cells that appeared more flattened out (arrows). (D) GPA transduced hFIB cultures grown in RA&EGF-containing SFM showed a further reduction in cell density and the presence of birefringent elongated (arrow) and smaller polygonal cells (arrowheads). Scale bar: 70μm.

**Fig 3:**
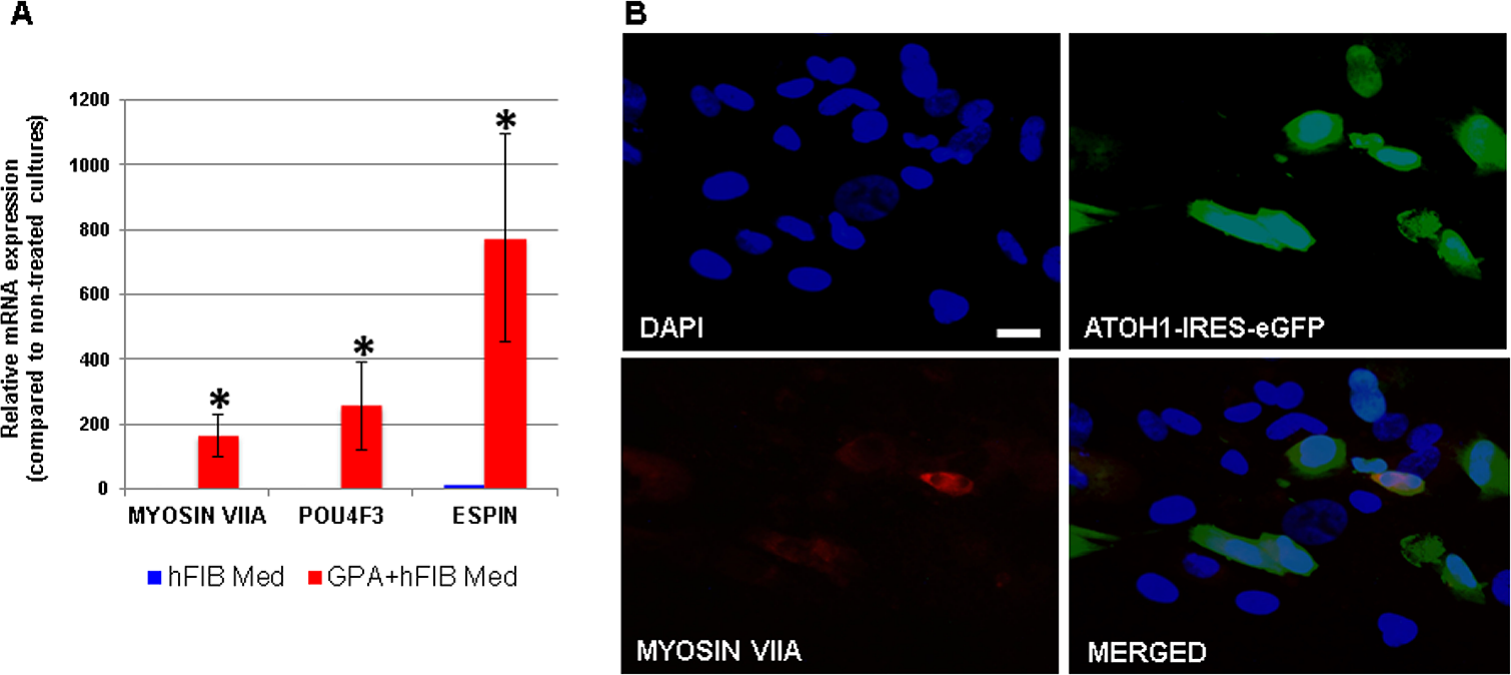
Induction of HC genes in GPA hFIBs grown in hFIB Med. (A) Relative mRNA expression of the endogenous genes *MYOSIN VIIA*, *POU4F3* and *ESPIN*, as measured by qPCR analysis. (B) Induction of MYOSIN VIIA protein expression in transduced (GFP-positive) hFIB cells. Note that due to the very high increase in mRNA levels in GPA-infected samples the low levels of mRNAs detected in control samples was barely visualized in the graph in (A). Asterisks: p-value<0.05; scale bar: 20μm.

**Fig 4:**
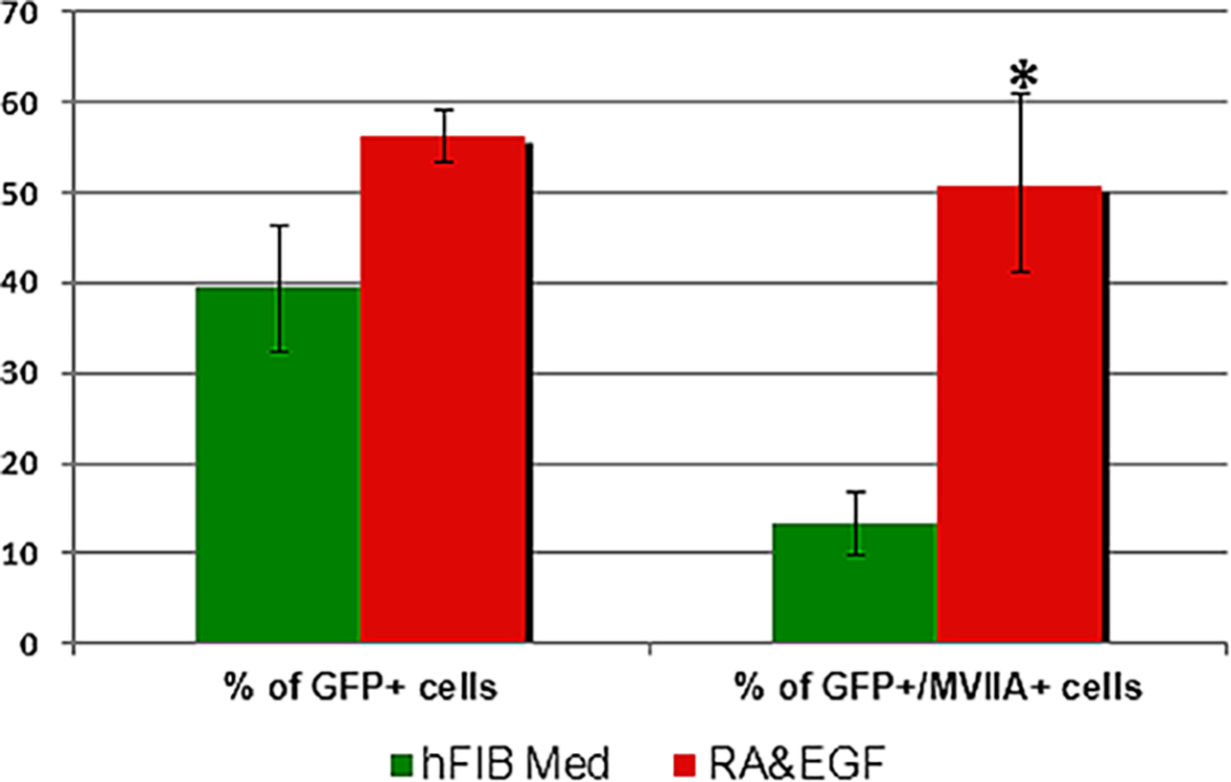
Quantification of the percentage of GFP- and Myosin VIIA colabelled cells. GPA hFIB cultures grown for two weeks in hFIB Med (green), and GPA hFIB cultures grown for two weeks in RA&EGF (red), following transduction. Asterisk: p value<0.005.

In a first set of experiments hFIBs were grown in standard fibroblast medium (hFIB Med) following infection. No apparent morphological changes were observed after two weeks (Fig 2A and 2B). To assess conversion towards the HC lineage we examined the expression of the HC markers *MYOSIN VIIA* and *ESPIN*, typically used for this purpose, together with endogenous expression of *POU4F3* via qPCR. This analysis revealed a significant increase in the mRNA levels of all three genes in the GPA hFIB cultures (Fig 3A). Unexpectedly, however, the high levels of *MYOSIN VIIA* mRNA were accompanied by a very weak induction of MYOSIN VIIA protein in a small percentage of the transduced cells, as revealed by immunocytochemistry (Fig 3B). Only 13,29±3,53% of the GFP-positive cells expressed MYOSIN VIIA protein (Fig 4).

### Culture of GPA transduced hFIBs in the presence of retinoic acid and EGF promotes the induction of HC genes and MYOSIN VIIA protein expression

**Fig 5:**
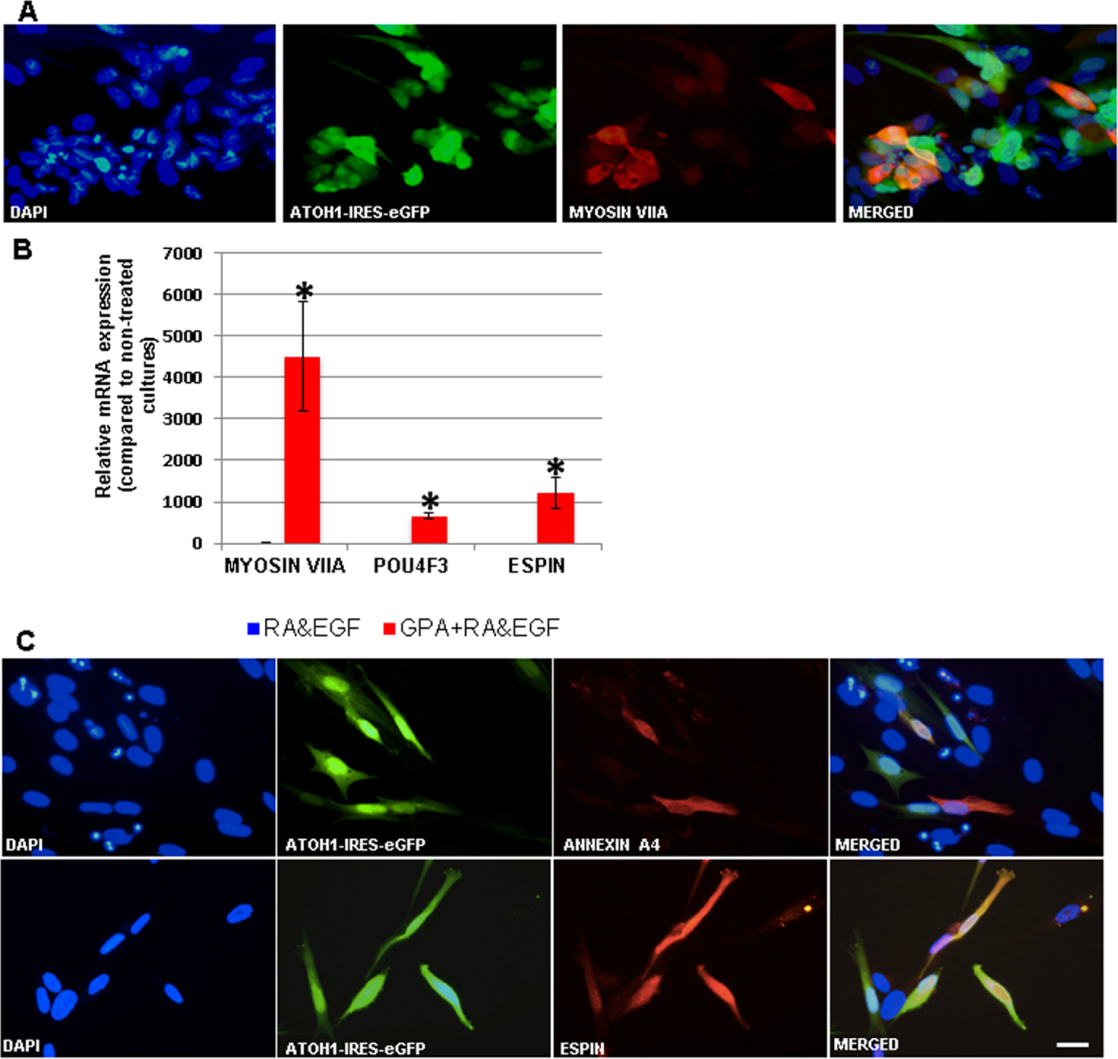
Induction of HC genes in GPA hFIBs grown for two weeks in RA&EGF. (A) Induction of MYOSIN VIIA protein expression visualized by immunocytochemistry. (B) Relative mRNA expression of the endogenous genes *MYOSIN VIIA*, *POU4F3* and *ESPIN*, as measured by qPCR analysis. (C) Induction of ANNEXIN A4 and ESPIN protein expression visualized by immunocytochemistry. Note that due to the very high increase in mRNA levels in GPA-infected samples the low levels of mRNAs detected in RA&EGF control samples were barely visualized in the graph in (B). Asterisks: p-value<0.05; scale bar: 20μm.

Previous studies have demonstrated that EGF and retinoic acid (RA) improve the differentiation of HC progenitors and the induction of Myosin VIIA protein [43,44,45]. Furthermore, Costa et al. [7] observed that addition of RA to GPA-expressing mESC cultures enhanced the efficiency of their programming protocol. With this in mind, we conducted a second set of experiments where GPA hFIBs were grown following infection in serum-free medium (SFM) containing both EGF and RA (Fig 5). Compared to GPA hFIB cultures maintained in hFIB Med, RA and EGF induced cells with increased birefringence (Fig 2C and 2D). These cultures also tended to contain a high proportion of GFP-positive cells (56,36±2,9% versus 39,51±7,00%, respectively) (Fig 4), although this difference was not statistically significant (p>0,06).

Immunocytochemistry revealed that a larger percentage of GFP-positive cells now expressed MYOSIN VIIA protein in cultures treated with RA and EGF compared to cells maintained in hFIB Med (50,95±9,87% in RA&EGF vs. 13,29±3,53% in hFIB Med; p<0,05) (compare Figs 5A with 3B). This was accompanied by very high levels of MYOSIN VIIA mRNA, well above those observed in control experiments, and those of GPA hFIBs grown in hFIB Med (p<0,005; Figs 5B and 3A). Compared to non-infected controls (grown either in hFib Med or in RA&EGF) expression of endogenous *POU4F3* and *ESPIN* mRNAs was also high in these experiments (Figs 3A and 5B). Additional immunocytochemical analysis was conducted in order to search for ANNEXIN A4 and ESPIN expression in the transduced cultures, both markers being typical of HCs [9]. Expression of ANNEXIN A4 occurred in 9,44±0,12% of the GFP-positive cells (Fig 5C). Despite the marked increase in *ESPIN* mRNA levels indicated by qPCR analysis (see above) we were unable to detect any polarized membrane projections that would indicate the formation of stereocilia, but rather a homogenous staining in a subset of the transduced cells (Fig 5C).

**Fig 6:**
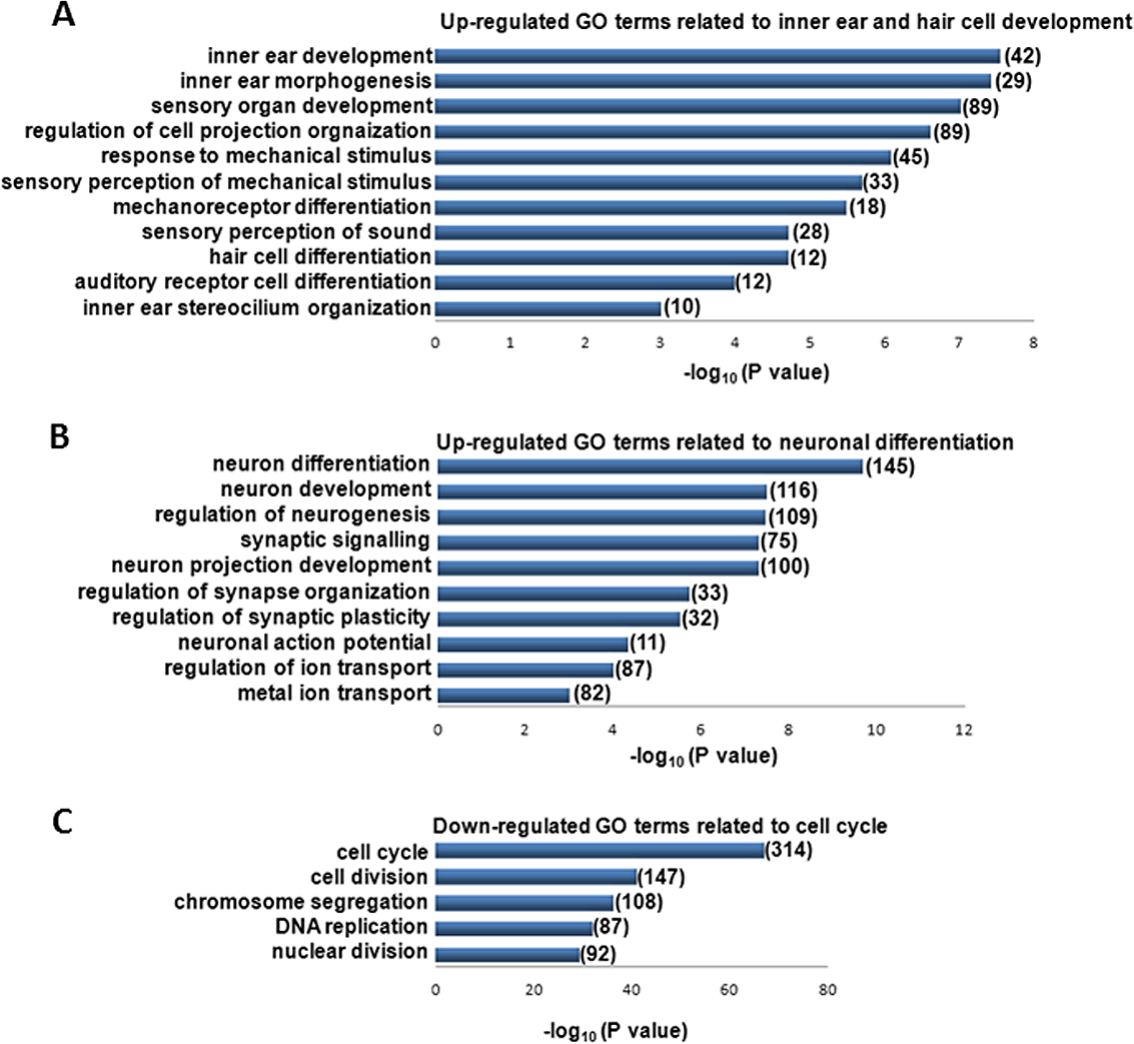
Gene ontology analysis. Performed using the PANTHER functional annotation tool for genes significantly up- or downregulated (fold change >2, P<0.01) in fibroblasts transduced with GPA and differentiated towards the HC lineage relative to undifferentiated fibroblasts. The number of upregulated genes included within each gene ontology functional term is shown.

In order to characterize the transcriptional profile of GPA hFIB cultures grown in RA&EGF, we carried out RNAseq analysis (see Methods). RNAseq from GPA hFIB cultures grown in RA&EGF was compared to that from non-treated control hFIBs cells (S1 and S2 Tables). Gene ontology analysis revealed an enrichment of genes related to HC development and differentiation, next to genes involved in neuronal differentiation in RA&EGF hFIBs (Fig 6A and 6B). This included genes involved in HC functions such as mechanoreception and sensory transduction. RNAseq analysis confirmed the upregulation of the hair cell markers *MYO VIIA* and *ESPIN*, next to *MYO15A/B*, *USH1C* and *CDH23* amongst others (S3 Table). Inner ear developmental genes that were up-regulated included *SOX9*, *BMP2/4*, *TGFB2*, *FGF9*, *NTN1*, *EPHB2 or PROX1* (S3 Table). Additionally, 94 genes that belong to the core group of HC-specific genes isolated from P1 mouse Atoh1-GFP-sorted hair populations were found to be upregulated in the transdifferentiated hFIBs [46]. Finally, consistent with the conversion of GPA-treated cultures, genes associated with cell cycle and cell division were found to be repressed (Fig 6C). Altogether these data indicate that the protocol based on GPA and RA/EGF treatment is able to initiate the conversion of human fibroblasts towards the HC lineage.

## Discussion

According to the latest estimates (March 2018) by the World Health Organization (WHO), disabling hearing loss affects 466 million people worldwide, and about one third of people over 65 years of age suffer from it (http://www.who.int/en/news-room/fact-sheets/detail/deafness-and-hearing-loss). It is the most prevalent sensorineural disorder in humans and its incidence is on the rise. The most frequent cause of hearing deficits is the loss of HCs, the mechanosensory receptors residing in the cochlea. There are just 15 thousand HCs in each cochlea, as compared to the millions of photoreceptors or olfactory receptors, and when they die there is no spontaneous regeneration. Although a very small number of supporting cells may behave as putative progenitor cells in post-natal mice, they do not seem to become active following damage to the adult mammalian cochlea and missing HCs are not replaced by new ones [1,2,29,47].

A large source of HCs and otic progenitors is necessary in order to provide a platform where to carry out extensive analyses on the molecular mechanisms governing the proliferation of otic progenitors, their differentiation and HC survival. The final aim of the research in the field is to identify a set of transcription factors, miRNAs or small molecule combinations that induce new HCs *in vivo*, to establish cultures of otic progenitors or HC-like cells amenable to analyses and to use them in future regenerative therapies. Accordingly, over the last few years, several groups reported the expansion of otic progenitors [6,48] and the differentiation of ESCs and iPSCs, of human and murine origin, to HC-like cells [6,7,8,9,10,11,49]. This is the first step in order to identify transcription factors and signalling pathways that can lead to regeneration in the inner ear, and thus the restoration of hearing.

Transplantation of progenitors or HC-like cells derived from exogenous cell types may be the only available option in some instances. For instance, when the lost HCs are replaced by a flat epithelium constituted by supporting cells that have become refractory to transdifferentiation, or when a genetic mutation is the underlying cause of the existing hearing deficits [49,50,51]. With respect to the latter, recent progress in iPSC and CRISPR technologies has rendered the *in vitro* correction of such mutations and the subsequent differentiation of the genetically corrected cells into HC-like cells a feasible option [49,52,53]. Nonetheless, the use of human ESCs or iPSCs as sources for HC-like cells raises a series of concerns. Next to ethical issues and the possibility of immune rejection, there is also the risk that ESCs or iPSCs that remain undifferentiated in the transplant may result in the development of teratomas [54]. In addition, despite the clear advantages posed by the advent of iPSC technology [15,19,49,52], such as the possibility of obtaining patient-derived HC-like cells that would not encounter any immune rejection response, obtaining true iPSC clones is costly and requires specific expertise [19], something that limits its widespread application.

It is in this setting that transdifferentiation of human somatic cells to HC-like cells may become an interesting alternative worthwhile to explore. In this work, we adopted a direct conversion approach whereby we obtained transdifferentiated cells from hFIB cultures that expressed a battery of HC genes, as evidenced by qPCR immunochemistry and RNAseq analysis. This was achieved through the over-expression of three transcription factors, *ATOH1*, *POU4F3* and *GFI1*, the former being considered a master regulatory gene in the HC lineage [55,56], while the latter two also play critical roles in HC survival [51,57,58,59]. Transient expression of this combination of genes in mESC cultures induces a very fast (4 days) conversion of over 50% of the ES cells into HC-like cells [7]. This is in contrast to the much longer duration of the protocols that have met with success when differentiating hESCs or hiPSCs [6,8,9,11]. In our experimental set-up we confirmed the expression of MYOSIN VIIA protein and *MYOSIN VIIA*, *POU4F3* and *ESPIN* mRNAs after 12 days of infection. Furthermore, preliminary experiments indicated that expression of these markers requires an even shorter time span, since high levels of MYOSIN VIIA protein expression are already observed at 7 days post-infection (data not shown). On the other hand, preliminary analysis also indicates that a co-expression of ATOH1 and GFI1 alone is already able to induce some MYOSIN VII A protein expression, while this is not the case when any of the factors is expressed by its own.

Despite transduction rates of above 50%, accompanied by the expression of MYOSIN VIIA protein in about half of the transduced GFP-positive cells, and the induction of other HC genes such as *POU4F3*, *ESPIN*, and *ANNEXIN A4*, our protocol faces a series of important limitations. Firstly, although our results provide evidence that conversion towards the hair cell lineage has been initiated, it is unclear if true reprogramming of the hFIB cells is taking place. This would require the confirmation of a stable phenotype of the transdifferentiated cells in the absence of the exogenous expression of GPA. Furthermore, each of the coding sequences for *GFI1*, *Pou4f3* and *ATOH1*, were cloned into separate lentiviral vectors. As a consequence, and although qPCR data and immunocytochemistry demonstrated the overexpression of each of the different transgenes, actual expression levels of the three transcription factors may vary from cell to cell, leading to differences in the extent of conversion within individual cells. Additionally, the use of lentiviral vectors conveys the integration of exogenous DNA sequences into the host genome, and the concomitant risk of insertional mutagenesis. This also ensures long-term expression of the transgene, while transdifferentiation to stable cellular phenotypes has often been correlated to a loss of transgene expression and the induction of the endogenous counterparts [26,49]. *ATOH1* expression, although necessary for the appearance of HCs in the inner ear, needs to be downregulated during HC maturation and its overexpression has been reported to result in cell death [60]. This effect, together with the absence of hair cell stereocilia, that could indicate further differentiation of the transduced cells [61], might be caused by the sustained expression of *ATOH1*. On the other hand, it is possible that our culture system lacks the appropriate conditions to sustain the survival and maturation towards transdifferentiating cells. Modification of these conditions might lead to better yields of more mature HC-like cells in our cultures [5,9].

Soon after transduction, cell density of GPA hFIB cultures grown in RA&EGF is reduced, and the observation of the cultured cells suggested a halt in cell proliferation. RNAseq analysis confirmed a downregulation of cell cycle genes very much like that of GPA-expressing mESCs [7]. Cell cycle arrest associated with the conversion of GPA hFIBs into post-mitotic cells may thus hinder the extensive characterization of these cells. For example, isolation of individual cell clones where to carry out more extensive analyses was not feasible, and whole transduced cultures had to be used for this work.

Several modifications will be potentially useful to improve our current protocols. For instance, inducible systems carrying the three ORFs together [7,30] and the use of non-integrative vectors may allow tight control of the expression of the transgenes, avoid the risk of insertional mutagenesis and, eventually, long-term expression of the transgenes [62,63]. Given the short period of time required for the conversion process, and the unsuitability of maintaining high levels of expression of the *ATOH1* gene, a transient overexpression of the GPA genes should suffice. In this regard, adenoviruses, as well as integrase-defective lentiviral vectors have already yielded successful outcomes [62,64,65,66]. Furthermore, there are other ways to guide cellular transdifferentiation, such as the introduction of DNA sequences via non-viral methods [67,68], or the use of proteins, microRNAs, RNAs or combinations of small molecules [17,69]. In order to improve cell survival and differentiation, several reports have shown that 3-dimensional culture settings promote self-organization into tissue-like structures and favour the emergence of mature phenotypes [5,8,9,14,70,71].

Further work may also improve the limited yield of differentiated cells obtained. Differentiation into proliferating otic progenitors would represent a clear advantage over the current model, both regarding the availability of cells for *in vitro* analysis and, more importantly, as an abundant source of cells for future regenerative therapies. Regarding this point, it has already been shown for other lineages that it is possible to obtain progenitor cells by the overexpression of key cell fate determination genes together with pluripotency genes [72,73,74].

In summary, our data validate the potential of the GPA combination to transdifferentiate human somatic cells into cells expressing hair cell markers. Direct conversion of somatic cells into another cell type reaches higher efficiencies when the starting and the sought-for cell populations present closer epigenomes [26]. Therefore, the ground is also set for *in vivo* studies to overexpress the GPA combination in the cochlea, where cell types such as the supporting cells are more closely lineage related to HCs, and where transdifferentiation should occur under the optimal environmental conditions. Successful *in vivo* transdifferentiation of cochlear cell types towards the hair cell fate following GPA overexpression in mature animals would lend support to its application as a possible avenue towards future regenerative therapies.

## Supporting information

S1 Table Upregulated genes in fibroblasts transduced with GPA and treated with RA and EGF. (XLSX)

S2 Table Downregulated genes in fibroblasts transduced with GPA and treated with RA and EGF. (XLSX)

S3 Table Genes upregulated in fibroblasts transduced with GPA and treated with RA and EGF related to inner ear development and sensory perception of sound. (XLSX)
