# Correction: Transcription factor induced conversion of human fibroblasts towards the hair cell lineage

**DOI:** 10.1371/journal.pone.0297736

**Published:** 2024-01-22

**Authors:** María Beatriz Duran Alonso, Iris Lopez Hernandez, Miguel Angel de la Fuente, Javier Garcia Sancho, Fernando Giraldez, Thomas Schimmang

There are errors in the Funding section. The correct Funding statement is: The project was funded by Junta de Castilla y Leon (Project VA024U16, Feder 2014–2020), Fundacion La Marato (Project 201227-30-31), Red de Terapia Celular and Red de medicina regenerativa y terapia celular de Castilla y Leon and Mineco (Project BFU2016-76580-P). The funders had no role in study design, data collection and analysis, decision to publish, or preparation of the manuscript.
